# Driving a motor vehicle and psychogenic nonepileptic seizures: ILAE Report by the Task Force on Psychogenic Nonepileptic Seizures

**DOI:** 10.1002/epi4.12408

**Published:** 2020-06-09

**Authors:** Ali A. Asadi‐Pooya, Timothy R. Nicholson, Susannah Pick, Gaston Baslet, Selim R. Benbadis, Massimiliano Beghi, Francesco Brigo, Jeffrey Buchhalter, Luciana D'Alessio, Barbara Dworetzky, David Gigineishvili, Richard A. Kanaan, Kasia Kozlowska, W. Curt LaFrance, Alexander Lehn, David L. Perez, Stoyan Popkirov, Chrisma Pretorius, Jerzy P. Szaflarski, Benjamin Tolchin, Kette Valente, Jon Stone, Markus Reuber

**Affiliations:** ^1^ Epilepsy Research Center Shiraz University of Medical Sciences Shiraz Iran; ^2^ Department of Neurology Jefferson Comprehensive Epilepsy Center Thomas Jefferson University Philadelphia PA USA; ^3^ Section of Cognitive Neuropsychiatry Institute of Psychiatry, Psychology and Neuroscience King's College London London UK; ^4^ Department of Psychiatry Brigham and Women's Hospital Harvard Medical School Boston MA USA; ^5^ Comprehensive Epilepsy Program University of South Florida and Tampa General Hospital Tampa FL USA; ^6^ Department of Mental Health AUSL Romagna Ravenna Italy; ^7^ Department of Neurology Franz Tappeiner Hospital Merano Italy; ^8^ Department of Pediatrics University of Calgary AB Canada; ^9^ Epilepsy Center Ramos Mejía y el Cruce Hospitals Buenos Aires University CONICET Buenos Aires Argentina; ^10^ Department of Neurology The Bromfield Epilepsy Center Brigham and Women's Hospital Harvard Medical School Boston MA USA; ^11^ Department of Neurology & Neurosurgery Tbilisi State University Tbilisi Georgia; ^12^ Department of Psychiatry University of Melbourne Austin Health Heidelberg Australia; ^13^ The Children's Hospital at Westmead Westmead Institute of Medical Research University of Sydney Medical School Sydney NSW Australia; ^14^ Rhode Island Hospital Brown University Providence RI USA; ^15^ Princess Alexandra Hospital Brisbane QLD Australia; ^16^ Functional Neurology Research Group Departments of Neurology and Psychiatry Massachusetts General Hospital Harvard Medical School Boston MA USA; ^17^ Department of Neurology University Hospital Knappschaftskrankenhaus Bochum Ruhr University Bochum Bochum Germany; ^18^ Department of Psychology Stellenbosch University Stellenbosch South Africa; ^19^ Department of Neurology and the UAB Epilepsy Center University of Alabama at Birmingham Birmingham AL USA; ^20^ Department of Neurology Yale Comprehensive Epilepsy Center Yale School of Medicine New Haven CT USA; ^21^ Laboratory of Clinical Neurophysiology Department of Psychiatry University of Sao Paulo Sao Paulo Brazil; ^22^ Centre for Clinical Brain Sciences University of Edinburgh Edinburgh UK; ^23^ Academic Department of Neurosciences University of Sheffield Royal Hallamshire Hospital Sheffield UK

**Keywords:** driving, nonepileptic, PNES, psychogenic, seizure

## Abstract

**Objectives:**

This International League Against Epilepsy (ILAE) Report: (a) summarizes the literature about “driving and psychogenic nonepileptic seizures (PNES)”; (b) presents the views of international experts; and (c) proposes an approach to assessing the ability of persons with PNES (PwPNES) to drive.

**Methods:**

Phase 1: Systematic literature review. Phase 2: Collection of international expert opinion using SurveyMonkey®. Experts included the members of the ILAE PNES Task Force and individuals with relevant publications since 2000. Phase 3: Joint analysis of the findings and refinement of conclusions by all participants using email. As an ILAE Report, the resulting text was reviewed by the Psychiatry Commission, the ILAE Task Force on Driving Guidelines, and Executive Committee.

**Results:**

Eight studies identified by the systematic review process failed to provide a firm evidence base for PNES‐related driving regulations, but suggest that most health professionals think restrictions are appropriate. Twenty‐six experts responded to the survey. Most held the view that decisions about driving privileges should consider individual patient and PNES characteristics and take account of whether permits are sought for private or commercial driving. Most felt that those with active PNES should not be allowed to drive unless certain criteria were met and that PNES should be thought of as “active” if the last psychogenic seizure had occurred within 6 months.

**Significance:**

Recommendations on whether PwPNES can drive should be made at the individual patient level. Until future research has determined the risk of accidents in PwPNES a proposed algorithm may guide decisions about driving advice.

**Key Points**

- There is a lack of high‐quality evidence characterizing accident risks of drivers with PNES
- Expert opinion holds that it is generally appropriate to recommend driving restrictions for individuals with active PNES
- Experts identified a number of PNES features that may be compatible with safe private (non‐commercial) driving

## 1 INTRODUCTION

Psychogenic nonepileptic seizures (PNES), also known as dissociative seizures/ attacks/ events/ episodes, are defined by their superficial semiological resemblance to epileptic seizures or syncope, although the manifestations of PNES are not explained by epileptic discharges or other readily observable physiological changes. Instead, most PNES are thought to be non‐volitional responses to internal or external triggers perceived as threatening or challenging.^1^, ^2^ While patients with PNES do not fit into a single category of the current international nosologies of mental disorders, most who are given this label fulfill the diagnostic criteria of Functional Neurological Symptom (Conversion) Disorder (DSM‐5) or Dissociative Seizure Disorder (ICD11).^3^ The incidence of PNES has been observed to be 1.4‐4.9/100 000/year and the prevalence estimated as up to 33 per 100 000 of the general population. As such, PNES are one of the three most common diagnoses made when patients present to seizure clinics. Given that this condition most commonly affects young adults,^1^ questions about driving a motor vehicle safely and PNES often arise in clinical practice.

Most patients diagnosed with PNES self‐report loss of responsiveness or loss of awareness in their events.^4^, ^5^ Self‐reported loss of responsiveness in PNES is significantly associated with self‐reported seizure‐related injuries [odds ratio (OR): 3.5; 95% confidence interval (CI): 1.4‐8.7].^4^ Drivers with neurological conditions have been found to be more likely to cause road traffic accidents compared to controls (OR: 5.2; 95% CI: 2.6‐10.3), as have drivers with psychiatric disorders (OR: 3.6; 95% CI: 1.9‐6.9).^6^ These observations mean that it is plausible that drivers with PNES could be at increased risk of causing driving‐related accidents. However, in the absence of data proving an increased PNES‐associated risk, compulsory driving suspension may be inappropriate, given that loss of driving privileges can have a major negative impact on patients' quality of life, ability to socialize, and socioeconomic status.^7^, ^8^, ^9^ The fundamental challenge lies in identifying an appropriate balance between the safety of patients with PNES and the public on the one hand, and the independence, autonomy, and quality of life of patients with PNES, on the other hand.

Given that there are currently no widely agreed practice guidelines on how to counsel patients with PNES on driving, our aims were to (a) review the literature about driving a motor vehicle and PNES; (b) gather the views of an international group of experts in the field on the issue of driving a motor vehicle and PNES; and, (c) summarize the findings and propose guidance on decisions about driving advice for individuals with PNES (single or recurrent) based on the majority views of the contributing experts.

## 2 METHODS

### 2.1 Phase 1 (systematic literature review)

We searched MEDLINE (accessed through PubMed) for articles published before July 5, 2019, that included any of the search terms selected (non‐epileptic seizures, nonepileptic seizures, pseudoseizures, non‐epileptic events, nonepileptic events, pseudoepileptic seizures, psychogenic seizures, psychogenic events, psychogenic non‐epileptic attacks, psychogenic nonepileptic attacks, psychogenic non‐epileptic episodes, psychogenic nonepileptic episodes, dissociative seizures, psychogenic non‐epileptic seizures, psychogenic nonepileptic seizures, PNES, and hysterical seizures) AND “driving” (Appendix 1), in their abstracts and titles. Review articles on PNES and articles not written in English were excluded. We selected the relevant articles by first screening titles and abstracts and subsequently reading in full any articles that appeared to contain information addressing the issue of driving in patients with PNES. We applied the same strategy to search abstracts, titles, and keywords using Scopus. We also searched PsycINFO (with “driving” in any field and the other keywords in the title). Duplicate and irrelevant articles were excluded, and the quality of the evidence was rated. Classes of evidence were categorized using the American Academy of Neurology's criteria for studies of causation (Appendix 2).^10^ Reporting complied with the PRISMA (Preferred Reporting Items for Systematic reviews and Meta‐Analyses) statement.^11^

### 2.2 Phase 2 (collection of expert opinions)

On July 11, 2019, we emailed a questionnaire (using SurveyMonkey^®^) designed by AAP, TN, SP, and MR (the first three and the last authors) to 50 international experts in this field (including these four authors). An email reminder was sent five days later. The experts were selected by the first three and the last two authors on the basis of meeting the following criteria: all members of the International League Against Epilepsy (ILAE)‐PNES Task Force, authors with five or more publications on PNES since 2000 (as the first author), authors of the previous original research articles on the topic of “driving and PNES”, and board members of the Functional Neurological Disorders Society (www.fndsociety.org) with a particular interest in this topic. The survey included 10 questions (Appendix 3): one question about professional qualifications (1), three questions on the participants' personal experience with the issue of interest (2, 4, and 9), five questions probing their opinions about the matter of interest (3, and 5‐8), and a final question about the respondent's interest in participation in the refinement process of the driving decision‐making guidance (10). The survey was closed on July 23, 2019. The first three and last two authors collected and analyzed the responses and developed a first draft of a summary based on the results of the review and the electronic survey (phases 1 and 2). A “majority opinion” was defined as one endorsed by more than 50% of the respondents.

### 2.3 Phase 3 (procedure for generating an expert opinion statement)

In a series of follow‐up email communications (total number of emails exchanged: 180), all participants who were interested in developing an opinion manuscript (n = 23) reviewed the initial draft. This draft included a report of the participants' responses to the survey. The draft was revised and improved by the panel through multiple rounds of email correspondence between the co‐authors.

In order to resolve issues that had arisen during this discussion, a second SurveyMonkey^®^ online questionnaire was created and distributed to the 23 participating experts (Appendix 4). A final opinion document with a flow diagram intended to support decision‐making processes relating to PNES and driving was prepared, underwent further revision, and was ultimately approved by all the participants in phase 3. There was no face‐to‐face meeting; the process was accomplished exclusively by email correspondence and draft revisions. This document underwent further review by the Psychiatry Commission, Task Force on Driving Regulations, Publication Council, and Executive Committee of the ILAE prior to submission of the manuscript for publication.

In thinking about an appropriate driving safety assessment paradigm, we deliberately focused on PNES. Clearly, decisions about driving privileges must take account of all aspects of the person's health, which could affect their ability to control a motor vehicle. This is particularly relevant in a condition such as PNES, which is commonly associated with comorbidities including epilepsy or other mental health disorders, and in which patients may take medications that could affect their ability to drive (such as sedative, antiepileptic, antidepressant, or antipsychotic drugs). Therefore, while features such as taking medication(s) that significantly impair driving ability and having active suicidal intent may be regarded as reasons for restricting driving privileges, these are not specific to PNES and are applicable to all patients with any medical or psychiatric condition. Importantly, the specific focus of the current paper on PNES means that the suggestions made here always need to be considered together with any additional driving restrictions related to other causes (such as epilepsy) which should—if appropriate—also be applied when decisions are made about a patient's fitness to drive. In any case, in which greater or more prolonged restrictions apply for other reasons than PNES, these more extensive driving restrictions should apply in full.

## 3 RESULTS

### 3.1 Phase 1

**FIGURE 1:**
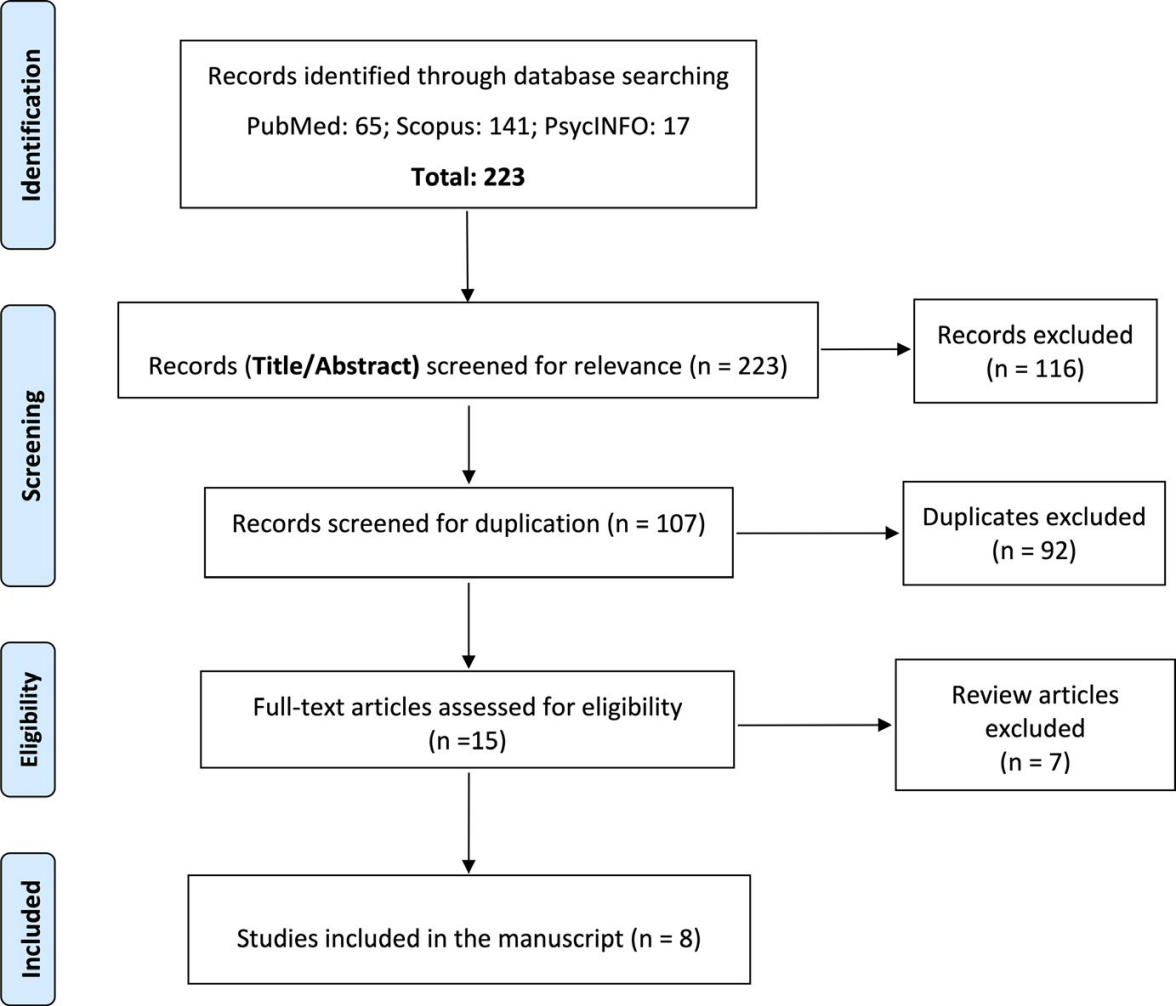
Preferred Reporting Items for Systematic Reviews and Meta‐Analyses (PRISMA) Flow Diagram of the study

The search strategy yielded eleven relevant articles in PubMed^7^, ^12^, ^13^, ^14^, ^15^, ^16^, ^17^, ^18^, ^19^, ^20^, ^21^ and four articles in Scopus^22^, ^23^, ^24^, ^25^ (three were reviews and not in English, and one was a review article in English). The PsycINFO database search did not yield any additional articles (Appendix 1 shows the details of the review process for each keyword in each of the search engines). Table 1 describes the methodology, main results, and quality ratings of the included articles. The manuscripts included comprised of seven surveys of patients and healthcare professionals and one cross‐sectional study. A study of 20 patients with PNES intended to assess whether they had a higher than average risk of car accidents.^19^ This small study did not find an increased risk of motor vehicle accidents among drivers with PNES; the accident rate in this population did not exceed that in the general population.^19^ The surveys highlight that there is controversy of opinions on driving regulations in previous studies. An additional seven narrative review articles were excluded (Figure 1). None of the eight research studies provided class I, II, or III evidence (Table 1).

**TABLE 1:** Details of the original research articles discussing driving in patients with psychogenic nonepileptic seizures (PNES)

1st author/Year/Country	Study type	Methods	Results	Class of evidence
Benbadis/ 2000/ USA	Survey & cross‐sectional study	Survey of 37 physician‐members of the American Epilepsy Society & study of a population of 20 patients with PNES, comparing driving records over a 5‐year period with general population accident rates.	49% of survey respondents applied the same restrictions for PNES as for epilepsy; 32% did not place patients with PNES under any restrictions; 19% decided on a case‐by‐case basis. The retrospective study of 20 PwPNES captured a total of 8 accidents, with no fatal crashes. The accident rate in this population did not exceed that in the general population.	IV
Specht/2009/Germany	Survey	Email survey of 34 German epileptologists.	Same restrictions as stipulated for patients with epilepsy endorsed by 32.4% of respondents; no restrictions at all by 0%; decision on an individualized basis by 67.6%.	IV
Morrison/2011/ UK	Survey	54 members of the Association of British Neurologists	68% of epilepsy specialists recommended driving restrictions for PNES. Two respondents reported patients with PNES‐related motor accidents.	IV
Sahaya/2012/ USA	Survey	Questionnaire study of 115 healthcare professionals exploring views about PNES	Only 15% of the participants favored unrestricted driving by patients with PNES in those with active seizure disorders.	IV
Jirsch/2015/ Canada	Cross‐sectional	Patients attending a rapid‐referral first seizure clinic were entered into the study if they held a valid driver's licence and were considered medically unfit to drive according to national guidelines for driving licensure due to having experienced a seizure or an unexplained episode of lost consciousness.	106 of 192 (55%) patients attending clinic met guideline criteria requiring driver fitness counseling. 89 patients (46%) were considered medically unfit to drive following initial specialist consultation. Among those considered medically unfit to drive, 73% were ultimately thought to have experienced an epileptic seizure and 27% a non‐epileptic event (eg, syncope, PNES).	IV
Vaidya‐Mathur/2016/ USA	Survey	141 patients with PNES completed a questionnaire to assess their socialization practices and perceived barriers to socialization.	Driving prohibition was the most commonly endorsed barrier to socializing.	IV
Mahmud/2017/ USA	Survey	41 neurologists were surveyed about the length of their recommended driving restrictions for patients with suspected seizures.	The majority recommended driving restriction of <12 mo for PNES.	IV
Farooq/2018/ USA	Survey	75 electronic questionnaires were sent to neurologists and family medicine physicians to assess their opinion regarding driving risk in PNES.	8.3% endorsed a belief that these patients should drive without restrictions. 93% felt having guidelines would help them assess the driving risk in this population.	IV

### 3.2 Phase 2

Of the 50 experts approached, 26 responded to the survey (response rate: 52%). Respondents were based across five continents and eleven countries around the world (Argentina, Australia, Brazil, Canada, Georgia, Germany, Iran, Italy, South Africa, UK, and USA). Table 2 describes the experiences of the expert participants with patients with PNES. Most of the respondents were neurologists and reported having extensive clinical experience with PNES (over 200 patients in their lifetime). Twenty‐three percent of the participants (n = 6) were aware of at least one patient under their care, who had had an accident related to PNES while driving. However, the total number of patients reported by these experts to have had PNES‐related driving accidents was only eleven, during years of their practice (the total number of patients with PNES seen by all experts contributing to this report is not known).

**TABLE 2:** Participants' experience with patients with psychogenic nonepileptic seizures (PNES)

	Number (%)
Discipline of the participants	Neurology: 14 (54) Psychiatry: 7 (27) Psychology: 3 (12) Double board (Neurology & Psychiatry): 2 (8)
Number of patients with PNES respondents have managed in their lifetime	More than 200 patients: 15 (58) 100‐200 patients: 4 (15) Less than 100 patients: 7 (27)
Are you aware of a patient with PNES under your care, who has had an accident while driving due to their PNES?	Yes: 6 (23) (1,1,2, 2, and 5 patients were reported by different experts; one participant did not specify a number) No: 20 (77)

Table 3 describes the opinions of the expert participants on different aspects of the issue of driving in patients with PNES. Only one expert (4%) endorsed the statement that “all patients with PNES should be allowed to drive without any conditions,” whereas 88% of the experts endorsed that “there should be restrictions on driving for some individuals with PNES”. In response to “how long do people need to be PNES‐free before they should not be considered to have active PNES?”, most experts (62%) endorsed a three‐ to twelve‐month period (mode: six months). Factors specific to PNES that should prompt the application of driving restrictions and that were endorsed by more than 50% of the experts included the following: loss of awareness/responsiveness with PNES, history of PNES‐related injuries, no auras or warnings or otherwise predictable seizures, and patients who want to be commercial drivers. Additional non–PNES‐specific factors endorsed by more than 50% of the experts as prompting the need for driving restrictions included the following: on medication(s) significantly impairing driving ability and active suicidal intent. Most experts believed that an exception from a PNES‐related driving prohibition could be made in the following circumstances: if PNES happen exclusively at times when the person *could not* be driving (eg, at night in bed) and if PNES are *always* preceded by a sufficiently long prodrome allowing the person to pull a car out of traffic and make themselves safe. The second survey addressing remaining controversial/ disputed topics that was sent to 23 experts was completed by all of the initial respondents. Table 4 summarizes the experts' opinions captured by this survey. Most experts thought that driving restrictions for those seeking commercial driving permits should be more restrictive than for those wanting to drive privately.

**TABLE 3:** Experts' opinions about driving and psychogenic nonepileptic seizures (PNES)

	Number (%)
Should all patients with PNES, with or without active psychogenic seizures, be allowed to drive without any conditions?	Yes: 1 (4) No: 21 (81) Not sure: 4 (15)
If all patients with PNES should be allowed to drive without any conditions, which of the following reasons lead you to this conclusion? (You may select both)	In my clinical experience, these patients do not have accidents driving: 1 (4) I'm not aware of any evidence that patients with PNES are at increased risk of driving accidents: 4 (15) Skipped: 21 (81)
If patients with PNES should not be allowed to drive, which of the following statements do you endorse?	There should be restrictions on driving for some individuals with PNES: 23 (88) None of the individuals with active PNES (Patients with seizures) should be allowed to drive: 2 (8) Skipped: 1 (4)
If individuals with PNES should not be allowed to drive, please specify your definition of “active PNES”: How long do people need to be PNES‐free before they should be considered not to have “active PNES”?	1 mo: 2 (8) 3 mo: 4 (15) 6 mo: 9 (35) 12 mo: 3 (12) 24 mo: 1 (4) More than 24 mo: 0 Skipped: 7 (27)
If there should be restrictions on driving for some individuals with PNES, which patients with PNES should not be allowed to drive? (You can select more than one choice)	Patients with loss of responsiveness (with preserved awareness) with their seizures: 16 (62) Patients with loss of consciousness with their seizures: 21 (81) Patients with memory gaps with or after their seizures: 7 (27) Patients with no auras, warnings or otherwise predictable seizures: 16 (62) Patients with prolonged seizures (more than 5 min): 7 (27) Patients with motor seizures: 9 (35) Patients with akinetic (dialeptic) seizures: 9 (35) Patients with history of PNES‐related injuries: 17 (65) Patients with daily seizures: 10 (38) Patients with weekly seizures: 9 (35) On medication(s) that significantly impair driving ability: 18 (69) Patients who want to be commercial drivers: 14 (54) Patients driving with passengers in the car: 7 (27) Patients with active suicidal intent: 16 (62) Skipped: 1 (4)
Are there any circumstances in which patients with PNES should be allowed to drive?	PNES happen exclusively at times when person not driving (eg, at night in bed): 19 (73) PNES are always triggered by a factor that would not be encountered in a car (eg, meeting the perpetrator of a crime against the person): 12 (46) PNES are preceded by a sufficiently long prodrome allowing the person to pull a car out of traffic and make themselves safe: 14 (56) Skipped: 3 (12)

**TABLE 4:** Experts' opinions about some controversial issues on driving and patients with psychogenic nonepileptic seizures (PNES) (the second survey)

	Number (%)
Are there some types of PNES which are compatible with driving?	Yes: 17 (74)^a^ No: 5 (22) Skipped: 1 (4)
Do you agree with this statement? “Patients with active PNES should not be able to get a commercial driving license for a longer period (> 12 mo), regardless of seizure semiology or illness features.”	Yes: 14 (61) No: 6 (26) Other: 3 (13)
For how long does an individual need to be PNES‐free before they can be considered fit to apply for a commercial driver's license?	18 mo: 3 (13) 2 y: 4 (17) 3 y: 3 (13) 5 y: 7 (30) Other: 5 (22) (two said 12 mo and three did not specify) Skipped: 1 (4)
What do you think about the length of time someone should have had seizures “only in situations when person would not be driving (eg, at night in bed)” before it is safe to allow them to drive?	6 mo: 3 (13) 12 mo: 13 (57) 2 y: 3 (13) 3 y: 0 5 y: 0 Others: 4 (17) (variable responses)
What do you mean by "only in situations when person not driving"?	Only at night in bed: 10 (45) Other: 13 (55) (various non‐driving situations such as at home or at school)

^a^ Examples include the following: minor motor or sensory PNES with preserved awareness and responsiveness and with no loss of motor control.

### 3.3 Phase 3

Email discussion between the participants of survey one yielded further clarifications of the proposal for an individualized driving safety assessment process:

The majority of the experts considered that individuals with active PNES should generally not be allowed to drive if any of the following criteria are met:

1. Loss of awareness/responsiveness with their psychogenic seizures
2. History of PNES‐related injuries
3. No auras or warnings or otherwise predictable psychogenic seizures
4. If PNES semiology suggests that ability to drive would be impaired during a psychogenic seizure.
5. Patients who want to be commercial drivers.

Post‐survey email discussions of point 4 yielded the following clarification: Examples would include convulsive or thrashing limb movements or visual disturbances. When it is uncertain whether the semiology of a psychogenic seizure would disrupt driving (eg, brief twitching of left shoulder), it is advisable only to consider the semiology as “not disruptive” if the patient has already had a typical psychogenic seizure while driving without experiencing any impairment in control of the vehicle or driving ability.

The majority of the experts considered that patients with active PNES can be allowed to drive *private (non‐commercial)* vehicles in the following circumstances even if any of the above‐mentioned criteria are met (active PNES per se is not considered a contraindication to driving):

1. If PNES happen exclusively at times when the person could not be driving (eg, at night in bed).
2. If psychogenic seizures occur exclusively after exposure to very specific triggers that they could not possibly encounter when driving (eg, confined spaces without windows, specific objects, or places acting as traumatic reminders).
3. If PNES are *always* preceded by a sufficiently long prodrome allowing the person to pull a car out of traffic and make themselves safe. Some patients report a recognizable warning sign (eg, light‐headedness and headache, etc), which invariably precedes each psychogenic seizure and would always provide enough time (minutes to hours) to safely bring the car to a halt. In all other cases, psychogenic seizure onset should be considered “sudden and unpredictable” when assessing a person's fitness to drive.

Importantly, these exemption criteria from the driving prohibition only apply if a patient has a well‐established and stable PNES pattern (ie, the pattern has been established for at least twelve months and there are no other reasons—including other types of seizures—why they should not be allowed to drive). These exemption criteria should not apply if the situational context or the semiology of the psychogenic seizures has varied in a relevant fashion.

### 3.4 Definition of “active PNES”

Initial responses to the question how long individuals should be considered to have “active PNES” after their last seizure ranged from three to twelve months. At the end of the discussion and refinement process (phase 3), the contributing experts settled, by majority views, on six months, the mode of their initial responses. While the uncertainty about this point reflects the lack of research on this issue, this means that the expert group viewed a period of six months free of PNES as a sufficiently long time to lift any restrictions to private driving imposed in relation to this disorder. However, especially reflecting the greater risk to the public associated with commercial driving, it was the view of the majority experts in this survey that individuals seeking a commercial driving license should have been free of PNES for a much longer period (two to five years) before restrictions could be removed, regardless of psychogenic seizure semiology or illness features, in order to clearly establish that they have overcome this condition.

**FIGURE 2:**
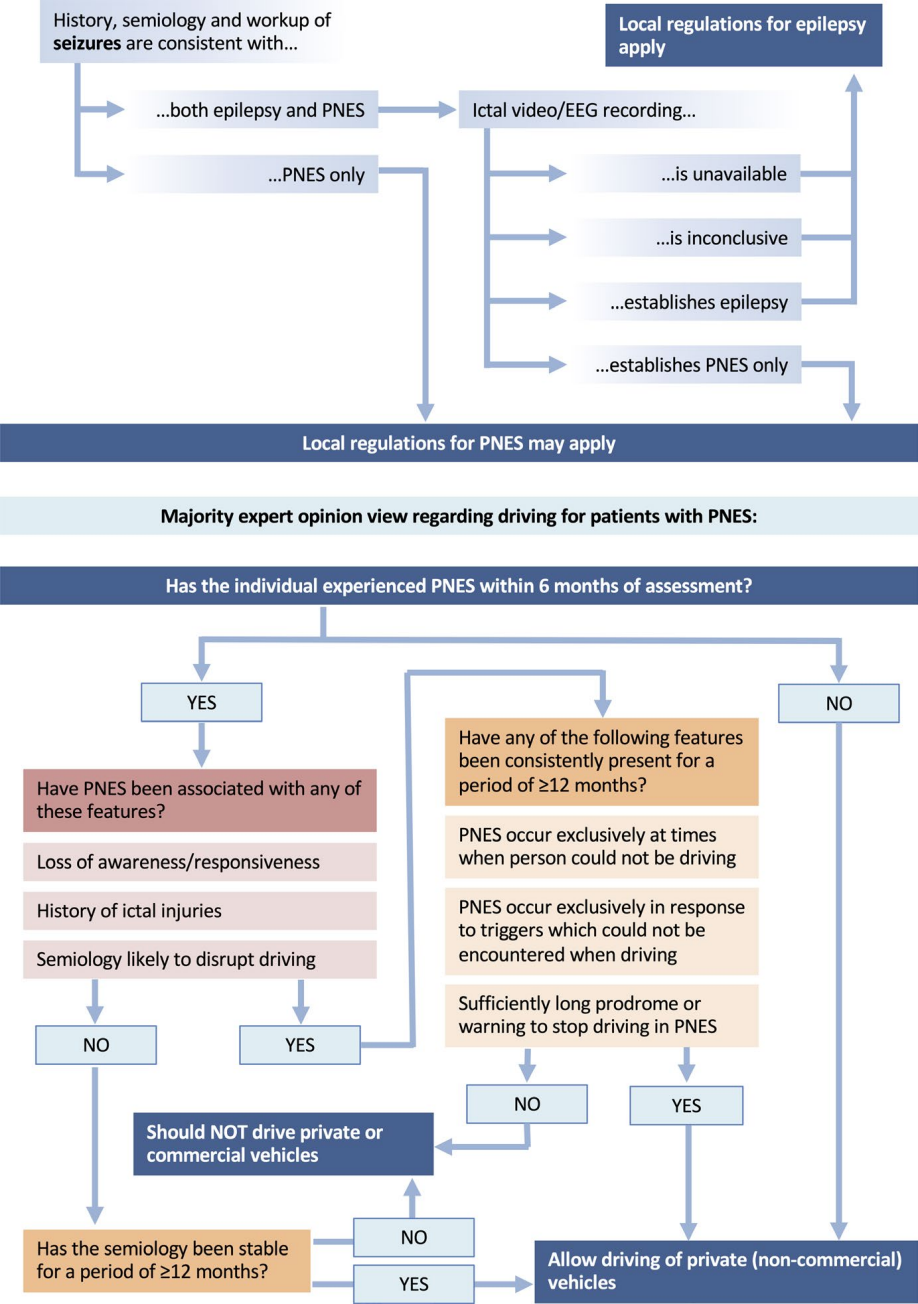
Expert proposal for a decision‐making process about the safety of patients with PNES to drive

Ultimately, the expert email discussion of the previous literature and current surveys yielded a flow diagram with a proposal for a decision‐making algorithm for assessments about driving safety in individuals with PNES (Figure 2).

## 4 DISCUSSION

While it is possible that some patients with PNES are more likely than other members of the general public to be involved in road traffic accidents, there is currently no compelling evidence supporting or refuting this proposition directly. One small study of 20 patients suggested that there was no increased risk of motor vehicle accidents among patients with PNES,^19^ but larger scale studies are clearly needed. In one survey of health professionals, over 90% of neurologists and family medicine physicians endorsed the necessity of guidelines for driving safety decisions in patients with PNES.^16^ In the absence of high‐quality evidence, an expert opinion statement providing preliminary guidance on this important social issue may be helpful. This survey and exchange of ideas documented variable practices across many epilepsy centers and provides basis for future explorations of this topic.

The nature of driving restrictions in relation to medical conditions and the question of whether these are necessary at all continues to be debated.^16^, ^17^, ^18^, ^19^, ^26^ In order to provide a sense of perspective, it may be helpful to consider the risk variability associated with demographic characteristics. For instance, it is recognized that the accident risk of male drivers under the age of 25 years is five to seven times greater than the mean accident risk, although this observation does not mean that members of this demographic group are banned from driving.^27^ However, in line with previous surveys focusing on PNES, the experts contributing to the present project held the view that driving restrictions should be recommended, at least for some individuals with this disorder. Despite the uncertainties and lack of evidence, it seems appropriate to err on the side of caution when driving privileges are concerned, especially given that the risks associated with driving would not just affect the individuals with PNES themselves. It is likely that the risks to others would be even greater in relation to commercial than private driving. It could therefore be argued that drivers with PNES should demonstrate to the community that they are safe to drive.^28^ Individuals affected might do so by having been PNES‐free for a period of time before being allowed to drive commercially.^28^ In relation to private (non‐commercial) driving, the panel of experts contributing to this study ultimately considered a six‐month PNES‐free period as reasonable evidence that a PNES disorder is under control. A considerably longer period of complete PNES control would be appropriate before commercial driving could be allowed.

The perception of PNES as a clinically heterogeneous disorder was reflected by the fact that the experts contributing to this project felt that, in certain circumstances, exceptions could be made from the general rule that patients with active PNES should not drive: The majority suggested that private (non‐commercial) driving should be permissible for individuals with PNES if there is a clearly established pattern of PNES happening exclusively at times when the person could not be driving, if PNES occur exclusively after exposure to very specific triggers that affected individuals could not possibly encounter when driving, and/or if the individuals always experience clear warning signs of sufficient duration that it would make it feasible for them to pull over their car and stop driving safely prior to an event.

The group of individuals with comorbid epilepsy and PNES presents a particular diagnostic and treatment challenge. While the overwhelming majority of patients with PNES do not suffer from epileptic seizures, a substantial minority (around 20%) have comorbid epilepsy.^29^, ^30^ As long as a patient with mixed epilepsy/PNES is experiencing epileptic seizures (or if the treating physician is not sure whether the epileptic seizures have stopped), then driving restrictions related to epilepsy should be invoked.^28^ Similarly, patients whose seizures are of uncertain etiology and who have not received a diagnosis of PNES that is sufficiently certain for the treating physician only to recommend treatment for this disorder and to stop any erroneously prescribed antiseizure medication treatments should be advised to adhere to laws restricting driving with epileptic seizures. However, if the treating physician firmly concludes that all ongoing seizures are due to PNES and that there have been no epileptic seizures for the period of time required by the relevant state law relating to epilepsy, then the opinions in this document would be applicable.

Changes in semiology represent another important challenge when exemptions from PNES‐related driving restrictions have been made. Although PNES semiology has been shown to be relatively stable over the short term,^31^ there is evidence that, over the longer term, it is more variable than the semiology of epileptic seizures.^32^, ^33^ This means that the suitability of individuals with PNES to drive needs to be reviewed at regular intervals. Further, those with active PNES who are allowed to drive on the basis of the proposed exception criteria need to be made aware that they must stop driving if the nature of their seizures changes and the criteria supporting their exception from driving prohibition are no longer met.

We acknowledge that our project has several limitations. The most important of these is the lack of sufficient data informing us of the risk of PNES in relation to driving. The wording of survey questions and other questions not addressed may have influenced the results. For example, our questions did not specify whether relevant ictal features should be patient‐reported, witness‐supported, or clinician‐observed—these specifications may have had an effect on the results. Only a limited number of experts were approached and only 52% of those participated, raising the possibility that the opinions reflected in this manuscript are not fully representative of wider expert opinion. Additionally, there could be patients to whom none of the semiological features listed to aid the assessment of driving fitness apply, and further clarification may be needed on driving recommendations for patients with additional functional neurological symptoms, addressing concerns regarding phenotypic evolution and the intersection of PNES and paroxysmal functional movement disorders. Some of the variability in the responses of the experts to our survey reflects the fact that there is no universally accepted definition of PNES. For instance, some experts hold that individuals may experience PNES with exclusively sensory symptoms (without impairment of awareness), while others would only use this diagnostic label if there was an associated impairment of consciousness or self‐control. If the risk of driving with PNES is similar to that observed in the general population, then presentations of expert opinion like this may, with hindsight, cause unnecessary harm and restriction to individuals with this diagnosis. Conversely, the opinions may be too optimistic and cause harm by underemphasizing the risks. Finally, some countries in the world may already have explicit driving regulations for individuals with PNES which supersede any recommendations made here in those countries [eg, in Australia, there are regulations for driving in “seizures and epilepsy,” without specific reference to PNES, which may be considered applicable to PNES, however.^34^ In the UK, patients with dissociative seizures (PNES) must not drive and must notify the Driver and Vehicle Licensing Agency (DVLA). Licensing may be considered when the driver or applicant has been event free for 3 months^35^].

However, until it is possible to develop evidence‐based guidelines based on well‐designed case‐control and prospective cohort studies, the present systematic review and synthesis of expert opinion represent a first step to the development of an individualized assessment procedure for patients with PNES wanting to drive. In order to achieve the goal of providing evidence‐based guidelines, treating physicians should collaborate with mental‐health professionals, motor vehicle licensing authorities, patient groups, caregivers, and others, to fully represent the multitude of relevant perspectives on this complex issue. We acknowledge in particular that, regardless of medical risk, driving regulations reflect societal pressures and legal responsibilities. We recognize that in response to a condition where an individual experiences recurrent and apparently unpredictable loss of consciousness, there may be a public demand for regulations that are similar to those for epilepsy, even if the associated risks were lower. In the absence of relevant evidence, expert opinion may empower clinicians to make the best possible decisions about driving restrictions in relation to PNES using the algorithm proposed in Figure 2 based on individual patient features.

## CONFLICT OF INTEREST

Ali A. Asadi‐Pooya received honoraria from Cobel Daruo, Raymand Rad, Tekaje; royalty: Oxford University Press (Book publication); and grants from the National Institute for Medical Research Development. Timothy Nicholson is funded by a UK National Institute of Health Research (NIHR) Clinician Scientist Award. The views expressed are those of the authors and not necessarily those of the NHS, the NIHR, or the Department of Health. Susannah Pick has no conflict of interest. Benjamin Tolchin has received research funding from a US Veteran Administration (VA)'s VISN1 Career Development Award; the VA Pain Research, Informatics, Multimorbidities, and Education (PRIME) Center of Innovation; and the CG Swebilius Trust. He has received honoraria from Columbia University Medical Center and the American Academy of Neurology. Selim Benbadis is consultant for Bioserenity (DigiTrace), Brain Sentinel, Cavion, Ceribell, Eisai, Greenwich, Growhealthy, LivaNova, Neuropace, SK biopharmaceuticals, Sunovion; speakers bureau for Eisai, Greenwich, LivaNova, Sunovion; member for Epilepsy Study Consortium; grant support from Cavion, LivaNova, Greenwich, SK biopharmaceuticals, Sunovion, Takeda, UCB; royalties as an author or Editor for Emedicine‐Medscape‐WebMD, UpToDate; and editorial Board for Emedicine‐Medscape‐WebMD, Epileptic Disorders, Epilepsy and Behavior, and Expert Review of Neurotherapeutics. Dr Dworetzky is a consultant for Digitrace and BestDoctors. She receives royalties from Oxford University Press (book publication), and from UCB for the Padsevonil trial. She is on the editorial board for Epilepsy Currents and is on the professional advisory board for the Epilepsy Foundation of New England. Dr Szaflarski has received research funding from National Institutes of Health, National Science Foundation, Department of Defense, Shor Foundation for Epilepsy Research, Eisai, Epilepsy Foundation of America, Food and Drug Administration, Serina Therapeutics, UCB Pharmaceuticals, Xenon Pharmaceuticals, Compumedics Neuroscan, Inc, State of Alabama, and the University of Alabama at Birmingham (UAB). He serves as an associate editor of the journals Journal of Epileptology and Epilepsy and Behavior Reports and on editorial boards of the journals Epilepsy & Behavior, Folia Medica Copernicana, and Journal of Medical Science and as a contributing editor for Epilepsy Currents. Dr Perez has received funding from the NIH and Sidney R. Baer Jr Foundation, and honoraria from Harvard Medical School, Movement Disorder Society, American Academy of Neurology, and Toronto Western Hospital. Stoyan Popkirov has received a speaking fee from Novartis. Dr Brigo received travel support from Eisai; he acted as consultant for Eisai, LivaNova, and UCB Pharma; he serves on the editorial board of the journal Epilepsy & Behavior and is Co‐Chair of the ILAE Guidelines Taskforce and editor of the Cochrane Epilepsy Group. Dr Stone reports personal fees from UptoDate, outside the submitted work; Dr Stone runs a self‐help Web site for patients with functional neurological disorders. www.neurosymptoms.org. It is free and has no advertising. Dr Stone carries out expert testimony work in personal injury and negligence cases involving functional disorders. Dr Baslet receives royalties from Oxford University Press. Dr LaFrance has served on the editorial boards of Epilepsia, Epilepsy & Behavior; Journal of Neurology, Neurosurgery and Psychiatry, and Journal of Neuropsychiatry and Clinical Neurosciences; receives editor's royalties from the publication of Gates and Rowan's Nonepileptic Seizures, 3rd ed. (Cambridge University Press, 2010) and 4th ed. (2018); author's royalties for Taking Control of Your Seizures: Workbook and Therapist Guide (Oxford University Press, 2015); has received research support from the Department of Defense (DoD W81XWH‐17‐0169), NIH (NINDS 5K23NS45902 [PI]), Providence VAMC, Center for Neurorestoration and Neurorehabilitation, Rhode Island Hospital, the American Epilepsy Society (AES), the Epilepsy Foundation (EF), Brown University and the Siravo Foundation; serves on the Epilepsy Foundation New England Professional Advisory Board; has received honoraria for the American Academy of Neurology Annual Meeting Annual Course; has served as a clinic development consultant at University of Colorado Denver, Cleveland Clinic, Spectrum Health and Emory University; and has provided medico‐legal expert testimony. Markus Reuber received educational grant from UCB, speaker fees from UCB, Eisai and LivaNova, and honoraria from Elsevier and Oxford University Press. Others have no conflict of interest. We confirm that we have read the Journal's position on issues involved in ethical publication and affirm that this report is consistent with those guidelines.

## APPENDIX 1

### The search keywords included “driving” and those in the first column of the table

**Table:**

Keywords (AND driving)	Medline (PubMed) (Title/Abstract)		Scopus (Title/Abstract/Keywords)		PsycINFO	
	Primary hints	Relevant articles	Primary hints	Relevant articles	Primary hints	Relevant articles
Non‐epileptic seizures	14	4	17	6 (5 duplicates)	2	1 (duplicate)
Nonepileptic seizures	6	5 (5 duplicates)	19	9 (9 duplicates)	3	2 (2 duplicates)
Pseudoseizures	2	1	9	4 (4 duplicates)	1	1 (duplicate)
Non‐epileptic events	2	1 (duplicate)	3	2 (2 duplicates)	0	0
Nonepileptic events	0	0	2	0	0	0
Pseudoepileptic seizures	1	0	2	1 (duplicate)	0	0
Psychogenic seizures	14	7 (2 duplicates)	24	11 (8 duplicates)	5	3 (3 duplicates)
Psychogenic events	2	1 (duplicate)	5	1 (duplicate)	0	0
Psychogenic non‐epileptic attacks	0	0	2	1 (duplicate)	0	0
Psychogenic nonepileptic attacks	0	0	2	0	0	0
Psychogenic non‐epileptic episodes	1	1 (duplicate)	1	1 (duplicate)	0	0
Psychogenic nonepileptic episodes	1	1 (duplicate)	3	2 (2 duplicates)	0	0
Dissociative seizures	0	0	4	1 (duplicate)	0	0
Psychogenic non‐epileptic seizures	7	3 (3 duplicates)	9	4 (4 duplicates)	2	1 (duplicate)
Psychogenic nonepileptic seizures	5	5 (5 duplicates)	16	10 (10 duplicates)	3	2 (2 duplicates)
PNES	10	7 (6 duplicates)	22	9 (9 duplicates)	1	1 (duplicate)
Hysterical seizures	0	0	1	0	0	0
**Total**	**65**	**34 (23 duplicates)**	**141**	**62 (58 duplicates)**	**17**	**11 (11 duplicates)**

## APPENDIX 2

### American Academy of Neurology criteria for classification of evidence in studies of causation^6^

**Table:**

Classification	Criteria
I	Prospective cohort study with all relevant confounders controlled, masked or objective outcome assessments, and a) ≤2 primary outcomes, b) clearly defined inclusion/exclusion criteria c) ≥80% study completion rate.
II	Retrospective cohort study or case‐control study meeting all other class I criteria.
III	Cohort study or case‐control study meeting all class I or II criteria except a, b, or c above.
IV	Studies not meeting Class I, II, or III criteria

## APPENDIX 3

### Driving in PNES

1. What is your discipline?

Neurology
Psychiatry
Psychology
Other (please specify)

2. How many patients with PNES have you seen and managed in your lifetime?

More than 200 patients
100 ‐ 200 patients
Less than 100 patients

3. Should ALL patients with PNES, with or without active seizures, be allowed to drive without any conditions?

Yes
No
Not sure

4. If all patients with PNES should be allowed to drive without any conditions, which of the following reasons lead you to this conclusion? (You may select both)

In my clinical experience these patients do not have accidents driving
I'm not aware of any evidence that patients with PNES are at increased risk of driving accidents
Other (please specify)

5. If patients with PNES should NOT be allowed to drive, which of the following statements do you endorse? (Select one)

There should be restrictions on driving for some individuals with PNES
None of the individuals with active PNES (Patients with seizures) should be allowed to drive

6. If individuals with active PNES should not be allowed to drive, please specify your definition of “active PNES”: How long do people need to be PNES free before they should not be considered to have “active PNES”?

1 month
3 months
6 months
12 months
24 months
More than 24 months
Other (please specify)

7. If there should be restrictions on driving for SOME individuals with PNES, which patients with PNES should not be allowed to drive? (You can select more than one choice)

Patients with loss of responsiveness (with preserved awareness) with their seizures
Patients with loss of consciousness with their seizures
Patients with memory gaps with or after their seizures
Patients with no auras, warnings or otherwise predictable seizures
Patients with prolonged seizures (more than 5 minutes)
Patients with motor seizures
Patients with akinetic (dialeptic) seizures
Patients with history of PNES‐related injuries
Patients with daily seizures
Patients with weekly seizures
On medication(s) that significantly impair driving ability
Patients who want to be commercial drivers
Patients driving with passengers in the car
Patients with active suicidal intent
Other (please specify)

8. Are there any circumstances in which patients with PNES should be allowed to drive?

PNES happen exclusively at times when person not driving (eg, at night in bed)
PNES semiology suggests that ability to drive would be unimpaired during a seizure (eg, NO loss of responsiveness)
PNES are always triggered by a factor that would not be encountered in a car (eg, meeting the perpetrator of a crime against the person)
PNES are preceded by a sufficiently long prodrome allowing the person to pull a car out of traffic and make themselves safe
Other (please specify)

9. Are you aware of a patient with PNES under your care, who has had an accident while driving due to their psychogenic seizures?

Yes
No

If yes, how many patients with documented diagnosis of PNES with accidents are you aware of?

10. If you are interested in participating in developing the expert opinion and manuscript and be a co‐author, please write your name and best email address below:

## APPENDIX 4

### Survey 2

Are there some types of PNES which are compatible with driving?

- Yes (Explain:…)
- No

Do you agree with this statement? “Patients with active PNES should not be able to get a commercial driving license for a longer period (> 12 months), regardless of seizure semiology or illness features.”

- Yes
- No
- Other (Explain:…)

For how long does an individual need to be PNES‐free before they can be considered fit to apply for a commercial driver's license?

- 18 months
- 2 years
- 3 years
- 5 years
- Other: …

What do you think about the length of the time someone should have had seizures 'only in situations when person not driving (eg, at night in bed)' before it is safe to allow them to drive?

- 6 months
- 12 months
- 2 years
- 3 years
- 5 years
- Others: …

What do you mean by "only in situations when person not driving"?

Only at night in bed
Other:
